# Life impact of ankle fractures: Qualitative analysis of patient and clinician experiences

**DOI:** 10.1186/1471-2474-13-224

**Published:** 2012-11-21

**Authors:** Steven M McPhail, Joel Dunstan, Julie Canning, Terry P Haines

**Affiliations:** 1 Centre for Functioning and Health Research, Metro South Health, Buranda Plaza, Corner Ipswich Road and Cornwall Street Buranda, Brisbane, Australia; 2 School of Public Health & Social Work and Institute of Health and Biomedical Innovation, Queensland University of Technology, Kelvin Grove, Brisbane, Australia; 3 Department of Physiotherapy, Princess Alexandra Hospital, Ipswich Road, Brisbane, Australia; 4 Department of Physiotherapy, Monash University, Frankston, Australia; 5 Southern Health, Allied Health Research Unit, Cheltenham, Australia

## Abstract

**Background:**

Ankle fractures are one of the more commonly occurring forms of trauma managed by orthopaedic teams worldwide. The impacts of these injuries are not restricted to pain and disability caused at the time of the incident, but may also result in long term physical, psychological, and social consequences. There are currently no ankle fracture specific patient-reported outcome measures with a robust content foundation. This investigation aimed to develop a thematic conceptual framework of life impacts following ankle fracture from the experiences of people who have suffered ankle fractures as well as the health professionals who treat them.

**Methods:**

A qualitative investigation was undertaken using in-depth semi-structured interviews with people (n=12) who had previously sustained an ankle fracture (patients) and health professionals (n=6) that treat people with ankle fractures. Interviews were audio-recorded and transcribed. Each phrase was individually coded and grouped in categories and aligned under emerging themes by two independent researchers.

**Results:**

Saturation occurred after 10 in-depth patient interviews. Time since injury for patients ranged from 6 weeks to more than 2 years. Experience of health professionals ranged from 1 year to 16 years working with people with ankle fractures. Health professionals included an Orthopaedic surgeon (1), physiotherapists (3), a podiatrist (1) and an occupational therapist (1). The emerging framework derived from patient data included eight themes (Physical, Psychological, Daily Living, Social, Occupational and Domestic, Financial, Aesthetic and Medication Taking). Health professional responses did not reveal any additional themes, but tended to focus on physical and occupational themes.

**Conclusions:**

The nature of life impact following ankle fractures can extend beyond short term pain and discomfort into many areas of life. The findings from this research have provided an empirically derived framework from which a condition-specific patient-reported outcome measure can be developed.

## Background

Traumatic musculoskeletal injuries are a common problem that may result in short or long term pain and disability [1-5]. Fractures around the ankle are one of the more commonly occurring forms of trauma managed by orthopaedic teams worldwide with Australasian data citing an incidence of 43.5 fractures per 100,000 persons per year [6-8]. Despite their high incidence, ankle fractures may be considered by some to be a ‘lesser’ injury in comparison to other fractures (such as multiple trauma, hip fractures or fractures of the axial skeleton) and have attracted less empirical research in comparison to other common fracture types [9-14]. This potential consideration of ankle fractures as a lesser injury may be due to a perception that ankle fractures are localised in nature and have a high success rate of fracture reduction and union with established treatment protocols [9,15]. However, any perception that ankle fractures have a low rate of sub-optimal outcome and negligible negative long term consequence are not founded in empirical data. Prior empirical research has indicated the impact of ankle fractures may not be restricted to pain and disability caused at the time of the incident but continue for an extended duration [9,15].

Long term effects of ankle fractures have been reported to include physical, psychological, and social consequences [9]. It has been reported that physical impairments following ankle fractures may include pain, functional impairment and the development of post-trauma arthritis [16]. Negative psychological consequences following ankle fractures have been reported to include fatigue, depression, anxiety and sleep disturbances [9]. Negative social consequences have included difficulty returning to work and dependence on disability benefits [9]. These types of negative consequences are comparable to those that have been reported among other severe fracture types [13,17-20].

There is some controversy as to the proportion of patients who recover well following ankle fractures [21]. Some previous studies have identified that 52% to 87% of patients have good to excellent clinical outcomes after an ankle fracture [7,22-25]. In contrast, a number of follow-up studies looking at patient outcomes between 14 months and 6 years following fracture have found that few patients reported a full recovery in most areas [8,9,26]. Specifically, 52% of patients had psychological complaints due to the initial injury, [9] and 52% had difficulties with sport activities [8]. Nilsson, Nyberg et al.[26] found that 51% self-report poor function with complaints of ongoing stiffness and swelling, pain with walking, and an impaired ability to climb stairs. A recent systematic review of long term outcomes from 1822 ankle fractures across 18 studies (4 to 14 years follow up) reported that approximately one in five did not result in a good or excellent outcome [15]. In these investigations, success was classified according to performance against a set of researcher-selected subjective symptoms and objective findings [15]. Additionally, measurement methodologies were frequently not described in detail or had not been tested for reliability and validity [15]. Insufficient or sub-optimal rehabilitation has been cited as a potential cause of long-term disability in this population [26]. However, a Cochrane systematic review of ankle fracture rehabilitation in adults highlighted that limited evidence is available at present to inform specific rehabilitation protocols for clinical practice [27].

One limiting factor when planning and conducting research among people with ankle fractures is the absence of a suitable ankle fracture specific patient-reported outcome measure. The inclusion of patient-reported outcomes as primary measures has become increasingly common across a wide range of clinical and research settings [28-33]. Common patient-reported outcomes that are frequently used among people with musculoskeletal conditions include measures of pain [34-36], physical function activity limitations [37-39] and health-related quality of life [40-45]. The use of patient-reported outcomes permit clinicians and health researchers to evaluate the effectiveness of an intervention based on the lived experience of the person with the condition under consideration [35,46-48].

Condition-specific patient-reported outcome measures should reflect those areas of life that are meaningfully influenced by the condition under consideration from the perspective of the patient [49,50]. The areas of life influenced by the condition may extend beyond physical functioning activity limitations [9]. This is in contrast to clinically derived measures that may focus on constructs that health professionals consider to be important (such as changes detected in x-ray images, joint range of motion or clinical performance tests) [51-53]. A condition-specific patient-reported outcome measure for use among ankle fracture patients during their rehabilitation should capture the effects of rehabilitation which patients (rather than health professionals) consider most important [49]. These effects must also be evaluated in a way that is valid, reliable and responsive to change over the entire rehabilitation period [54,55].

Investigations of ankle fracture rehabilitation included in a Cochrane systematic review of ankle fracture rehabilitation focused on clinical outcomes; including ankle range of motion and performance tests [27,56]. Some investigations used patient-reported outcomes to assess health professional defined physical activity limitations [57-62]. The most frequently used patient-reported outcome for this purpose was the Olerud Molander Ankle Scale [27,63]. This scale was reported by Olerud and Molander in 1984 to improve the way ankle symptoms were evaluated [63]. The scale includes nine parameters focusing on physical symptoms and physical activities (walking, stiffness, swelling, stair-climbing, running, jumping, squatting, physical supports, and work capacity) [63]. The scale includes two to five multiple choice response options for each parameter which the authors of the scale assigned a value of 0, 5, 10, 15, 20 or 25 (maximum total score is 100) [63]. While this scale is practical and represented advancement beyond describing ankle symptoms into overall subjective categories such as a ‘good’ or ‘poor’ outcome, the scale has been criticised for lacking a methodologically robust foundation with content and scores based on expert opinion alone [47]. There is also a marked lack of empirical evidence reporting favourable psychometric and clinimetric properties for this scale [47,55,63,64].

Absence of a robust content foundation or empirical evidence indicating favourable clinimetric properties is also a shortcoming of other patient-reported outcomes for the foot and ankle [47,57-59,62,64]. Other patient-reported measures identified in the Cochrane review of ankle rehabilitation included the Clinical Demerit Points (based on the Weber Protocol) [62], Lower Extremity Functional Scale (LEFS) [60,65], Inflammatory Score [61], Maryland Foot Score [59], a visual analogue scale [58] and a grading scale by Mazure in 1979 [57]. These measures lack a methodologically robust foundation for evaluating life impacts experienced by ankle fracture patients during their rehabilitation [47,49]. Their content and scoring are commonly based on expert opinion alone and tend to focus on physical symptoms or activity performance. With the exception of the LEFS, these measures also lack empirical support for key elements of validity, reliability and responsiveness [47,57-59,62,64].

The LEFS has demonstrated favourable clinimetric properties in non-ankle fracture populations [65-67], and during the acute phase of ankle fracture recovery [68]. However, the ceiling effect observed after the acute phase of ankle fracture rehabilitation is detrimental to its use as a primary outcome measure throughout the entirety of the rehabilitation process [68]. Additionally, the content (and subsequent scoring) of the LEFS focuses heavily on elements of performance related to physical tasks (including walking, squatting, running, standing, stairs, hopping) [65]. This is not necessarily a weakness for an instrument intended to assess patients’ ratings of their lower extremity physical function. The LEFS has a solid foundation of empirical data supporting its use for this purpose [65,67,69]. However, the LEFS was not developed with an empirical foundation for use as an ankle fracture condition-specific patient-reported outcome measure intended to evaluate the life impacts (including non-physical impacts) that are most meaningful to patients recovering from an ankle fracture [9].

In summary, a range of patient-reported outcomes have been used among people with ankle fractures. These measures frequently have some methodological foundation in previous examinations of particular aspects of validity and reliability. However, ideally patient-reported outcomes should have foundation in patient-reported impacts, in addition to performing well in studies reporting aspects of validity, reliability and responsiveness to change. Including the patient’s perspectives when evaluating ankle fracture interventions may be problematic in the absence of a condition-specific patient-reported outcome measure empirically derived from lived patient experiences [49]. Existing foot and ankle outcomes were designed to evaluate physical symptoms and activity performance across a range of lower limb conditions [47,63-65]. These measures are unlikely to capture the most salient physical and non-physical impacts experienced by people recovering from ankle fractures [9].

This study aimed to investigate the nature of life impacts following ankle fractures with the intention of describing a thematic conceptual framework based on these lived experiences of people who have suffered ankle fractures. The investigators considered the description of this thematic framework as a critical first step in the development of an ankle fracture specific, patient-reported outcome measure suitable for evaluating the impact of an ankle fracture on patients’ lives. The development of such a measure into a questionnaire format would likely permit efficient and effective assessment of the impact of ankle fractures on patients’ health-related quality of life (not just their physical activity limitations). A questionnaire for this purpose would have application in both clinical and research settings. This measure could have potential use at a single assessment or as a repeated measure to evaluate recovery (or decline) longitudinally. This would allow use in both observational and intervention studies; including clinical trials evaluating the effectiveness of ankle fracture rehabilitation protocols. Therefore, the purpose of this study was to not only investigate the nature of life impacts in the acute post-injury phase of recovery following ankle fractures, but to include life impacts across the recovery continuum and returning to work and usual daily living.

## Methods

### Design

Qualitative analyses of semi-structured interviews were undertaken. This design was selected as an appropriate modality to explore the range of perceived life impacts following ankle fractures founded on the lived experiences of patients who have suffered this injury.

### Participants and setting

The targeted inclusion of patients from across the temporal breadth of recovery was employed to promote the inclusion of a diversity of life impacts across the entire rehabilitation period. To this end, purposive sampling of adults who had experienced an ankle fracture (patients) and health professionals who have experience treating patients with ankle fractures were recruited from a tertiary hospital facility. Patient participants were recruited to fill three strata (ratio of 1:1:1) of time since ankle fracture from one to six months, from six months to two years and greater than two years. The investigators considered but decided against purposive sampling to ensure a range of fracture severities (in addition to temporal diversity) as prior research revealed negative consequences following ankle fracture were not necessarily dependent on the initial severity of fracture [15].

Health professionals from multiple discipline backgrounds were also interviewed to capture a range of health professional perspectives. While examining the opinion of health professionals was not the primary purpose of this investigation, the inclusion of health professionals who have seen many patients with ankle fractures was considered a feasible approach to add additional strength to the investigation. It was considered that health professionals may have been more likely to report an infrequent but severe life impact given their greater exposure to ankle fracture cases in comparison to patient participants. The authors considered that an echoing of some patient-reported life impacts by health professionals, without the addition of new categories or themes would lend weight to the reliability and validity of a thematic framework developed from patient responses. Purposive sampling was undertaken to ensure representation from four health professions who commonly make contributions to the management and rehabilitation of ankle fracture patients (orthopaedic surgery, physiotherapy, occupational therapy and podiatry). The investigators considered that the inclusion of at least one health professional from each of the four disciplines would be likely to add an additional degree of rigour to the development of the thematic framework.

### Recruitment

Patient participants were recruited from flyer advertisements posted within the hospital facility where the study was conducted. Health professional participants were recruited from similar flyers posted in orthopaedic clinic locations, staff notice boards and promotion of the research at a clinical staff meeting. The flyers contained a brief description of the study and a contact details for a member of the investigative team. This team member explained the study to potential participants in detail, provided a study information and consent form, and answered any questions relating to the study.

### Interview content and procedure

Consenting participants completed a single semi-structured interview in a private office or clinical consultation room at the hospital. Interviews were audio-recorded and transcribed verbatim (pseudonym names were inserted and patient participant responses were coded with a unique identifying number to protect participant confidentiality). Audio files and transcripts were stored digitally. The duration of interviews ranged between 32 and 65 minutes. The same member of the research team conducted all patient interviews. This interviewer was a physiotherapist who had extensive experience working with orthopaedic patients in clinical settings (although was not directly involved in clinical management of any participants in this study).

The interview content for patients included four broad partitions; 1) demographic and injury description, 2) life impacts immediately following the ankle fracture, 3) life impacts at approximately 6 weeks post-fracture (or immediately after the cast was removed), and 4) life impacts at the present time. During the demographics and injury description, participants were asked to describe the injury (including when the injury had occurred), as well as a description of the treatment they received. This included whether or not surgery to stabilise the ankle fracture was performed and the nature of any post fracture rehabilitation undertaken. This information was collected to describe the sample and is presented in Table 1.

**Table 1:** Demographic and fracture information from participants who suffered an ankle fracture

**Participant code**	**Gender**	**Age**	**How ankle fracture occurred**	**Bones fractured**	**Any other injury at time of fracture**	**Surgical fixation of ankle fracture**	**Weeks participating in rehabilitation therapies**	**Time since injury strata**
P1	Female	58	Tripped over pet dog	Distal fibula	no	None	4	> 24 months
P2	Male	38	Car accident (car versus car)	Distal tibia and distal fibula	no	ORIF*	8	> 24 months
P3	Male	19	Dropped while ‘crowd surfing’	Distal fibula	no	ORIF	4	Between 6 and 24 months
P4	Male	30	Motor bike accident (bike versus car)	Distal tibia and distal fibula	Haematoma adjacent to ipsilateral knee (resolved spontaneously)	ORIF	13	Between 6 and 24 months
P5	Female	49	Ankle twist injury playing sport (netball)	Distal fibula	no	None	0	> 24 months
P6	Female	24	Hiking (slip and fall)	Distal fibula	no	None	6	> 24 months
P7	Female	45	Fell off horse	Distal tibia and distal fibula	no	ORIF	5	< 6 months
P8	Female	47	Fall walking in platform shoes on uneven surface	Distal fibula	no	ORIF	4	< 6 months
P9	Female	28	Skiing (collision and fall)	Distal fibula	no	None	11	< 6 months
P10	Male	32	Motor bike accident (clipped curb on side of road)	Distal tibia and distal fibula	no	ORIF	7	< 6 months
P11	Male	29	Tackled playing football (soccer)	Distal tibia and distal fibula	no	ORIF	0	Between 6 and 24 months
P12	Male	23	Wakeboarding injury	Distal fibula	no	None	3	Between 6 and 24 months

*Open Reduction Internal Fixation (ORIF).

For the life impact portions of the interview (temporal partitions 2 to 4 outlined above), the investigators considered it possible that the interviewer’s clinical physiotherapy background may have impacted on the content being unintentionally focused on physical impairments and biomechanical functioning. For this reason an interview schedule was constructed to ensure non-physical impacts were discussed and explored. An example of the stimulus questions from one temporal partition is displayed in Table 2. This same pattern of stimulus questions was used in each of the three partitions (2 to 4), but with reference to different stages of recovery following rehabilitation as appropriate. In addition to these initial stimulus questions, the interviewer was instructed to use probing questions to explore the nature of all types of impacts that the patient began to describe in response to the initial scheduled questions. The interview schedule was initially developed from prior research reporting potential negative consequences of ankle fractures and the research teams own experiences [9]. Additionally, the interview schedule was able to be adjusted in response to data from earlier interviews. However, only one minor refinement, the addition of a question about impact on life roles, was added after the first three interviews.

**Table 2:** Example stimulus questions from a single partition of the semi-structured interview

**Question number**	**Stimulus question**
1	How was your ankle at the time?
2	How did that make you feel?
3	Were there any things that concerned you about your ankle when you were using it?
4	How did your ankle affect your ability to complete everyday activities around your house?
5	How did your ankle affect your ability to complete your occupation?
6	How did your ankle affect your ability to complete your leisure activities?
7	How did your ankle fracture change the types or amounts of activities that you actually participated or previously participated in?

A different researcher conducted the health professional interviews. This researcher had not worked as a colleague in clinical settings with any of the health professionals and was not in a dependent professional relationship (e.g. superior-subordinate) with any health professional participants. The same interview structure was followed for the health professional participants.

### Analysis

Qualitative analysis of interview transcripts for people who had experienced an ankle fracture was undertaken using thematic analysis [70,71]. Each phrase was coded and sorted into categories by two independent researchers. The categories were then grouped together with other related or similar categories. These emerging groups of related categories were then considered as an overarching theme that was subsequently described (based on the content of the categories and nature of the relationship between them). To support the rigour of this process, each of the two coders completed this task independently for each participant before meeting to compare their coding and emerging framework. A third independent researcher was available to mediate any unresolved coding disagreement between the two primary coders (for categories or emerging themes); however, no such disagreement occurred.

When a single phrase contained multiple life impacts that aligned with one or more categories, each relevant component was coded separately and grouped appropriately. Data saturation was considered to have occurred when 2 consecutive patient interviews did not add any additional categories or themes to the emerging framework. After the thematic framework was developed from patient data (Table 3), this coding process was repeated for the transcripts of health professionals who treat people with ankle fractures. However, the categories from health professionals were aligned under the thematic framework developed from the responses of people who had experienced an ankle fracture (where appropriate). New categories or themes from the health professionals’ responses (not yet represented) could be added to the emerging framework as indicated.

**Table 3:** Thematic conceptual framework of life impacts following ankle fractures, including categories represented within each theme

**Physical**	**Psychological**	**Daily living**	**Social**	**Occupational or Domestic**	**Financial**	**Aesthetic**	**Medication Taking**
*Physical impacts experienced*	*Psychological impacts experienced*	*Impacts on daily living activities*	*Social impacts experienced*	*Impacts on occupational or domestic tasks*	*Financial impacts experienced*	*Aesthetic impacts experienced*	*Experiences associated with medications*
1. Pain, ache, soreness or discomfort	1. Feelings of anxiety	1. Reduced participation in preferred recreation or leisure activities	1. Negative impact on relationship with spouse or significant other	1. Difficulty participating in usual work activity	1. Reduced income	1. Changed physical appearance due to weight gain.	1. Medication usage (including associated side effects)
2. Swelling	2. Feelings of depression	2. Reduced participation in health and fitness activities	2. Increased dependence on others in household	2. Difficulty completing household tasks	2. Use of savings	2. Now wear non-preferred footwear	
3. Decreased strength	3. Feelings of frustration	3. Difficulty participating in personal care activities (including showering and dressing)	3. Negative impact on personal relationships with family or friends.		3. Reduced discretionary spending		
4. Decreased range of movement/stiffness	4. Feelings of tiredness or fatigue	4. Difficulty sleeping			4. Increased cost of living (including healthcare costs)		
5. Altered sensation							
6. Difficulty walking (including flat surfaces, slopes and steps)							

Within each theme, the prominent emerging category or categories were identified based on the frequency and nature of responses (with many similar or related responses indicating a primary emerging category). These prominent categories for life impacts following ankle fractures are discussed in the text of the results section separately for patients and health professionals.

### Ethics

This investigation conformed to the Declaration of Helsinki and local legislation. The investigation was approved by the Princess Alexandra Hospital human research ethics committee and undertaken within the bounds of national ethical guidelines.

## Results

Thirteen patients and six health professionals were invited to participate in the investigation after responding to the study advertisement (and being deemed appropriate in meeting one of the required purposive sampling strata). However, one patient was not able to find a suitable time to schedule an interview, so declined participation. Twelve patient interviews and six health professional interviews were undertaken and included in analysis. Patient demographic and clinical information, including a brief summary of patients’ descriptions of their ankle fractures are presented in Table 1. The sample included diverse causes of ankle fracture and patient ages. There was equal gender representation. Seven patients reported receiving surgical stabilisation (each of these descriptions consistent with open reduction, internal fixation). Health professionals included an Orthopaedic surgeon (1), physiotherapists (3), a podiatrist (1) and an occupational therapist (1). Experience of health professionals ranged from 1 year to 16 years working with people with ankle fractures.

Data saturation occurred after ten patient interviews; with the final two patient interviews not contributing any further themes or categories. Data saturation being reached after only ten interviews may have occurred due to inclusion of patients who had experienced diverse and severe impacts following their ankle fracture. The rich and somewhat exhaustive data contributed by these patients covered a large proportion of categories included in the final thematic framework. The final framework is presented in Table 3. The eight emerging themes included Physical, Psychological, Daily Living, Social, Occupational and Domestic, Financial, Aesthetic and Medication Taking. The six health professional interviews were coded to this framework (Table 3) without addition of any further themes or categories. Phrases from the health professional participants were most frequently aligned under categories in the Physical or Occupational theme; few were aligned under the Psychological, Aesthetic or Social themes. Example quotes for each of the eight themes are also presented in Table 4. These quotes were selected being as representative of the overarching theme and diversity of life impacts across the patient sample.

**Table 4:** Examples of participant quotes from each theme in the conceptual framework

**Physical**	**Psychological**	**Daily living**	**Social**	**Occupational and Domestic**	**Financial**	**Aesthetic**	**Medication Taking**
*Physical impacts experienced*	*Psychological impacts experienced*	*Impacts on daily living activities*	*Social impacts experienced*	*Impacts on occupational or domestic tasks*	*Financial impacts experienced*	*Aesthetic impacts experienced*	*Experiences associated with medications*
“Very sore. Pain was the number one problem.”p1	“I was scared I was going to break it again…”p6	“No leisure. No Sports. Nothing.”p12	“Personal life is restricted with relationships…”p9	“It causes me pain… but it doesn’t stop me from working, but I am in more pain.”p6	“I was out of work so it affected money.”p11	“I haven’t been able to wear any of my high heel shoes.”p8	“It causes me pain- have to take medication…”p12
“Mainly if I stand on it too much, the ankle gets quite irritated and very swollen.”p2	“I felt anxious about putting weight on it… even after the doctor said I could.”p9	“I wasn’t able to do any running…”p5	“(My wife) and I ended up arguing…”p2	“… I commenced on light duties at work.”p2	“I had a fairly good deposit for a house… over the past 12 months I have slowly eaten away my savings account.”p10	“The only problem I had was just fitting into shoes… I wanted to wear high heel shoes to my daughters wedding…”p1	“the pills make me feel sick”p6
“It was weak because one leg was skinnier than the other… it felt like it might give way.”p12	“Emotionally I was affected. I used to feel quite depressed and down a lot…”p10	“Could not do anything. Could not even walk the dog, too painful.”p1	“… virtually can’t socialize anymore.”p3	“I didn’t do much around the house. I couldn’t house clean.”p5	“…and I had to keep paying for those tablets they had me on.”p4	“I put on 5-6kg by just sitting around… I was restricted by being stuck on my bed…”p4	“… I had to start taking sleeping tablets”p2
“… I was very limited in my (ankle) movements”p4	“it took so long… it was very frustrating”p4	“I don’t go to soccer any more. At all. I haven’t been there.”p11	“Your social life is affected in a way that you don’t want to go out…”p6	“If it was sore, I didn’t want to go to (work), so I didn’t go”p8	“I can’t date because I can’t get out and about and I can’t afford to date.”p12	“I have put on weight as a result of the fracture.”p6	“I needed some pain meds for a few weeks, but then it was fine”p5
“I could only walk for a certain amount of time before it hurt.”p5	“It was more effort to do anything, I felt so tired all the time…”p8	“I took on a lesser role… I modified the activity so it was less demanding on the ankle…”p2	“My daughter got annoyed (with me) cause I needed her to drive me…”p1	“I have had to change my job entirely because of my ankle injury… I am now not doing anything.”p10	“… anything that I want to do outside of sitting in the lounge room costs me money I don’t have.”p3	“… I now wear these silly slip-ons (shoes) everywhere.”p7	“…the tablets at the start made me sleep a lot”p2

### Theme 1: physical

A broad range of physical impacts were described by both patients and health professionals. These impacts included mechanical elements (swelling, reduced muscle strength, decreased range of motion) and associated afferent impacts (pain, discomfort, altered sensation etc.). Responses that described difficulty with walking without reference to any specific occupational or daily living impact were also grouped into this theme. For example, one participant noted “*I am limited on how far (and) fast… I can walk*” (p6). Impacts in this theme were not limited to the immediate post-fracture period: “*it was (many) months before I got the movement back*” (p10).

Pain was the primary emerging category reported by patients in this theme. Patient participants reported “*pain was the number one problem*” (p1). Immediately after the fracture and following the removal of the plaster, participants recalled substantial pain in their ankle. The present level of pain amongst the patient participants (of varying duration since injury) was heterogeneous with some reporting constant or daily pain; others described how the pain *“just got better over time”* (p5)*.* Swelling was also identified as a major concern for patients, particularly immediately following plaster removal.

Health professional responses were focused within the physical impacts theme more than any other. Health professionals identified pain as a primary impact of the ankle fracture that may not resolve after the initial post-fracture period. One health professional stated most of the patients are usually “*feeling quite a bit of pain*” (hp1) during the early post-fracture period another stated *“…pain is still a problem months after the cast (is) off for some (patients)” (hp4).* Health professionals were generally more articulate with their responses in this theme than patients. One health professional noted *“…restricted dorsi flexion is usually a problem”* (hp4) not only identifying restricted range of movement, but also commenting on a specific direction of movement commonly affected. Health professionals reported that swelling will be present, can be persistent and often causes discomfort.

### Theme 2: psychological

Participants reported a range of psychological and emotional impacts attributed to their ankle fracture that were grouped into this theme. These included depression, anxiety, frustration and tiredness or fatigue. Responses grouped into this theme were often described in relation to another impact. For example *“I was just so frustrated (that) it ached no matter what I did,”*(p10) and *“…ongoing pain just wore me out… I felt tired all the time*” (p8).

The severity of psychological impacts reported by patients was not consistent across respondents. Responses from patients about feelings of depression ranged from being “*…at an all time low in my life…*” (p10) to “…*it is a bit depressing”* (p2). Most participants stated that negative feelings resolved as they were able to return to activities undertaken prior to their ankle fracture. However, some participants reported ongoing unresolved anxiety or depression months after plaster removal. A participant who had experienced a difficult recovery after fracturing his ankle 18 months earlier stated he tended to *“feel quite depressed and down a lot, and dwell on what happened and keep replaying things in (his) mind”* (p10).

Health professionals infrequently described impacts that were aligned in this theme. Despite fewer responses in this theme, the impacts described were consistent with those described by patients. One health professional noted *“…some get a bit depressed about their situation”* (hp5). Another recalled working with patients who had become anxious about the risk of re-fracturing the ankle despite x-ray evidence of sound healing and reassurance from the health professional that fracture recurrence was not likely.

### Theme 3. daily living

The primary emerging category in the daily living theme was the impact on participation in preferred recreation or leisure activities. Participants reported many of the recreational and leisure activities they had participated in prior to the ankle fracture were not possible or had to be modified following the ankle fracture. Both patients and health professionals reported substantial impacts on health and fitness activities. Impacts on personal care tasks were generally described in the context of the early post fracture period, as were difficulties with sleeping.

Many patient participants stated they could still do aspects of their previous activities after sustaining the ankle fracture, but needed to alter these as a result of the fracture. For example one participant was still able to go swimming, but was not able to use a flipper since the fracture. Another participant stated “*I modified the activities I do so it’s less demanding on the ankle but still enjoyable*’ (p2). Some had stopped participating in recreation or fitness related activities altogether due to ankle fracture reporting they felt they “*couldn’t do anything*” (p12). Patient participants reported their ability to go walking or running was decreased, with one participant stating he was still unable to return to jogging almost a year post ankle fracture. Another stated *“I can now only go walking (for) about one and half kilometres… I used to walk 5km (regularly)”* (p7).

Health professionals described a range of impacts in this theme that were congruent with responses from patients. They similarly reported substantial impacts on health and fitness activities as well as a range of impacts grouped into the other three categories in this theme (Table 3). However, at least one health professional felt that some patients continued to avoid physical activity long after there was any anatomical indication to do so. They reported this often occurred despite reassurance that it was not only safe, but beneficial to return to living a healthy active lifestyle. The health professionals also commented that many patients have unrealistic expectations about how quickly their ankle will heal and they will be able to return to their usual activities. With one stating “*patients often think their ankle will get better quicker than it does*” (hp2).

### Theme 4: social

Impacts that were grouped within the social theme were diverse. In summary, they included the ability to undertake informal social activities with friends or family as well as reduced participation in formal social gatherings. Reports of the impact of their ankle fracture on ability to socialize differed across participants. Some stated that they had felt a decreased ability to be able to socialize with friends whereas as one patient stated they could still “*hang out with people*” (p3) and they actually spent more time with their friends than prior to their ankle fracture.

Some patients reported that they could not participate in many social activities so there was no point attending social gatherings. One participant reported staying home so she could keep her foot elevated instead of seeing friends. A number of participants felt they were a burden to friends and family. One patient stated he “*felt like (he) let (his) wife down*” (p2) and that it had negatively impacted their relationship. Participants reported they did not want to be “*a drag or pain*” (p1) to their friends or family.

Health professionals infrequently reported responses in this theme. They did however note the increased burden for family members who may be required to assist older adults who have fractured their ankle and have difficulty with certain tasks. One health professional also noted that some patients resist becoming dependent on others in their household, even for a short period, while others seem more than happy for this to occur.

### Theme 5: occupational or domestic

Both patients and health professionals reported impacts on a range of tasks included in this theme. These tasks included those involved with maintaining a household, paid employment or volunteer work. Some impacts reported in this theme were short lived and resolved after the initial post-fracture recovery and return to gait without the use of walking aids. Other impacts were long lasting and resulted in a career change for some.

The most frequent impact reported by patients was the need for light or modified duties at work and a reduction in home duties. Light or modified tasks at work had a major impact on most participants. Many reported that they “*…could not work fulltime”* (p1) and were restricted in their ability to work in the immediate post-fracture period. Many participants stated that everyday domestic activities were very difficult to do and that many “*didn’t do much housework*” (p9). Some participants who had returned to normal gait reported they were currently experiencing no limitation with occupational and domestic tasks stating their ankle is “*no problem*” (p5) and “*has no effect at all*” (p9) on current occupational and domestic tasks. In contrast, another participant stated *“I did not work for eleven and a half months… I ceased (my occupation) at that time”* (p10). That participant had subsequently changed occupations to a less physically demanding role.

The severity of impact on work activities reported by health professionals varied widely. Health professionals reported that some patients had changed occupations after the ankle fracture due to the specific physical demands of their role. One health professional noted this seemed to happen more often when the patient worked in a *“manual labour intensive”* (hp6) role in the immediate pre-fracture period. Health professionals reported that impacts on occupational and domestic roles were usually short-lived and most people returned to pre-fracture roles within the first few months following the fracture.

### Theme 6: financial impact

Responses in this theme included the financial impacts that were directly attributed to the ankle fracture. Responses focused on reduced income due to time off work, reduced work hours or an altered work role. An associated impact was the use of savings to compensate for reduced income or greater expenses (including expenditure on healthcare costs).

Patients reported financial impacts of mixed severity. Participants frequently reported reduced income as the primary financial impact. Many participants stated they were “*out of work so it affected money”* (p11) or suffered “*loss of income as (I was) unable to work at full capacity for some months*” (p2). However, patients also reported financial impacts that included reliance on savings and being forced to reduce their discretionary spending to compensate for the reduced income.

Health professionals frequently described impacts on paid employment activities. However, they did not extend this to include a description of personal financial implications.

### Theme 7: aesthetic impact

Responses grouped into the aesthetic theme included those that related to physical appearance, rather than function or other health attributes. Two distinct categories emerged in this theme; weight gain and having to wear non preferred footwear. Some responses about weight gain were discussed by patients in the context of health and fitness (and grouped into the health and fitness category), rather than under the Aesthetic Impact theme.

Patients reported impacts from footwear limitations and concerns with their appearance following weight gain they had attributed to the ankle fracture. Limitations with footwear were identified by almost all the female patient participants, and this was usually connected to the inability to wear high heels. One participant stated “*I wanted to wear high heel shoes to my daughters wedding… so I got quite upset when… (I) couldn’t wear high heels to (my) daughter’s wedding*” (p1). Footwear comments were not limited to female participants or to high heels. One male participant reported “I couldn’t just wear flip flops (uncovered recreational footwear).” A number of patient participants reported impacts on their appearance or clothing choices due weight gain since fracturing their ankle. One participant reported *“I was (a little) bit concerned about how I looked…”*(p6) after she “…*had put weight on”* (p6).

Health professionals described weight gain as an impact experienced by some patients as a result of reduced physical activity following their ankle fracture. However, this was raised in the context of health and fitness rather than an aesthetic impact. Health professionals infrequently identified difficulty for their patients in wearing preferred footwear in the immediate post ankle fracture period. It was stated by one health professional that those patients who suffered an ankle fracture “*may not be able to wear high heeled shoes*” (hp1).

### Theme 8: medication taking

Comments in this theme included impacts relating to medication usage. Both patients and health professionals infrequently reported responses that were grouped into this category. Descriptions of life impacts reported in this theme tended to be focused on pain medication usage in the first few months after the ankle fracture and plaster removal. However, some comments grouped into this theme were also mentioned in the context of sleeping (Table 3).

## Discussion

### Main finding

This investigation has successfully indentified a broad range of life impacts reported from the lived experiences of people who have suffered an ankle fracture. It has been the first to report an empirically derived thematic framework highlighting that the impacts of ankle fractures extend beyond short term pain and discomfort. The eight themes included in the framework were Physical, Psychological, Daily Living, Social, Occupation and Domestic, Financial, Aesthetic and Medication Taking (Table 3) impacts. Objective measures of impairment commonly used in clinical assessments for people with ankle fractures match life impacts reported in the Physical theme (including joint range of motion, pain and swelling) [57-62]. The framework developed from this study suggests patient-reported life impacts follow ankle fractures extend beyond physical impairment into many facets of life. Constructs identified by this study that are frequently not represented in existing instruments used among people with ankle fractures include those relating to Psychological, Social, Financial, Aesthetic and Medicine Taking impacts [57-62]. A patient-reported outcome measure that assesses these wider impacts would be a pragmatic approach for assessing broad impacts and subsequent recovery after ankle fractures.

The nature of life impacts described by health professionals were congruent with those reported by patients. However, health professionals’ responses focussed on physical and occupational impacts. This is not surprising given the nature of their routine interactions with people with ankle fractures. These interactions frequently focus on physical impairments, acute management and rehabilitation to return to daily living, occupational and domestic activities. Health professionals in this study also demonstrated some awareness of the broader impacts represented across the framework (Table 3), but did not discuss them in the same detail or with the same frequency as patient participants. Use of a patient-reported outcome that evaluates these broader life impacts may assist health professionals and researchers to gain a deeper understanding of patients’ recovery and identify when intervention for an associated life impact may be warranted (such as anxiety or depression) [72].

This investigation was an explorative study to formulate a framework of life impacts following ankle fractures. The study was not intended to investigate prevalence, prediction rules or associations between each of the life impacts. However, from participant responses it is clear that the severity and duration of life impacts reported varied widely across participants and themes. This variation is likely due to a range of factors including initial post-fracture management, anatomical alignment (or misalignment), post stabilisation rehabilitation and other personal attributes. It is also likely that the severity and duration of physical impacts are associated with other areas of life dependent on normal ankle functioning (such as routine health and fitness activities). These postulations are worthy of empirical research in order to justify whether more holistic interventions for people with ankle fractures are warranted.

There are two immediate implications from this study for the clinical management of patients with ankle fractures that are worthy of further consideration. First, multi-disciplinary care may be warranted in some cases. This is perhaps more likely to be true when the outcome of initial management was sub-optimal. Patients who end up suffering from diverse negative life impacts (depression, unemployment, chronic musculoskeletal pain, domestic difficulties etc.) may benefit from multi-disciplinary input in order to improve their recovery and optimise their health-related quality of life. Second, health professionals caring for patients recovering from ankle fractures should consider whether a change in their own practice is required in order to mitigate the risk of some impacts (e.g. chronic pain) that may be able to be addressed within available treatment options. Another consideration may be whether there is scope to improve communication between health professionals and patients regarding the likely pathway and timeline of recovery. This may also include discussion of active patient participation in their recovery through therapies or return to work programs. However, in the absence of clinical trials establishing the efficacy of various post ankle fracture rehabilitation protocols, it is difficult to draw further firm clinical implications or recommend practice change based on the qualitative data generated in this study.

Comparison to prior research is difficult as this has been the first qualitative study of this nature undertaken amongst people with ankle fractures. However, findings from this research are consistent with previous reports of physical impairments (including pain and functional impairment), psychological consequences (including depression and anxiety) and negative social consequences (including difficulty returning to work) among people who had experienced ankle fractures [9]. These types of negative consequences are also comparable to those that have been reported among other severe fracture types and painful musculoskeletal disorders [13,17-20]. Findings from this research are also consistent with previous investigations that have indicated incomplete recovery can affect patients for long periods of time after acute fracture stabilisation and bone union has occurred [8,9,26].

### Strengths and limitations

There are several limitations and strengths to this research. First, the investigation included a purposive sample of participants from a single geographical metropolitan location in a developed nation with high quality public healthcare services. People without access to similar healthcare services may have had different types and severity of life impacts following an ankle fracture than participants in this investigation. However, the inclusion of participants with a range of time since injury from early post fracture to greater than two years has also helped ensure representation of impacts across the continuum of recovery. This is also likely to have mitigated the potential influence of recall bias on the subsequent framework development by including patients who have recent memories of impacts at each stage of the recovery [73].

The patient interviews were conducted by a single researcher with a clinical background. This researcher’s clinical background may have inadvertently influenced the topic and nature of the interview. However, the semi-structured interview design provided some protection from this potential source of bias. The thematic analysis was also conducted by researchers familiar with common socio-demographic and clinical themes who were also aware of the potential for this framework to be used as a foundation for the subsequent development of a patient-reported outcome measure. This may have inadvertently influenced the way data was coding into categories and the grouping of categories into themes. However, the use of two independent coders, with a third researcher available to arbitrate any unresolved disagreement helped protect against this risk.

The inclusion of health professionals who have seen many patients with ankle fractures has provided additional strength to the investigation. The inclusion of only one health professional from three of the four included health disciplines may be considered a weakness of the investigation. However, on the other hand, health professionals were not the focus of this investigation, but were included to lend weight to (or refute) the validity of the thematic framework developed from patient responses. The health professionals that were included may have been more likely than patients to report an infrequent but severe life impact given their greater exposure to ankle fracture cases in comparison to patient participants. The investigators considered that the echoing of patient-reported life impacts by health professionals, without the addition of new categories based on health professional experiences, did lend weight to the thematic framework that was developed from patient responses (Table 3).

### Future research

A high priority for future research is the development and validation of an ankle fracture condition-specific outcome measure. The thematic framework from this investigation will be used as stimulus material for a questionnaire framework from which specific question items and response formats could be operationalised using a staged Delphi panel process or other suitable method. The content of this measure may include questions with Likert or multiple choice response options that allow patients to report the severity of impact (or absence of impact) across the categories and themes reported in the framework. Findings from this study indicate questions to be included in this measure should extend beyond physical impacts alone and include the broad range of impacts presented in the thematic framework (Table 3). Pending appropriate pretesting and psychometric evaluation, this outcome measure could have potential use as a self-completed questionnaire suitable for use in a variety of clinical and research settings; particularly to evaluate the success (or otherwise) of rehabilitation following ankle fractures. The breadth of impacts assessed may result in this measure having potential utility for indicating when a referral to an additional health discipline (or multi-disciplinary team) is warranted.

Future research should also include investigations to examine the nature of relationships between anatomical structures included in the ankle fracture and the nature of life impacts experienced, as well as the association between physical impairment routinely evaluated in clinical practice and the broader life impacts (such as depression). This line of research is likely to indicate whether more holistic interventions are required, or whether current models of care focusing on restoration of ankle physical functioning are adequate.

## Conclusions

The nature of life impact following ankle fractures can extend beyond short term pain and discomfort into many areas of life. The findings from this research have provided an empirically derived framework from which a condition-specific patient-reported outcome measure can be developed.

## Competing interests

The authors declare they have no competing interests.

## Authors contributions

SM contributed to research idea conception, data analysis and manuscript preparation, as well as manuscript review, appraisal and editing. JD and JC contributed to data analysis and manuscript preparation. TH contributed to research idea conception, data analysis as well as manuscript review, appraisal and editing. All authors read and approved the final manuscript.

## Pre-publication history

The pre-publication history for this paper can be accessed here:

http://www.biomedcentral.com/1471-2474/13/224/prepub
